# Designing theranostic radiopharmaceutical trials for uterine cervix cancer

**DOI:** 10.3389/fonc.2025.1708720

**Published:** 2026-01-14

**Authors:** Denise Fabian, Molly E. Buffington, Brianna S. Cagle, Steve M. Keefe, Michael K. Schultz, Charles A. Kunos

**Affiliations:** 1 Department of Radiation Medicine, University of Kentucky, Lexington, KY, United States; 2 Department of Pharmacy Services, University of Kentucky, Lexington, KY, United States; 3 Department of Pharmaceutical Sciences and Experimental Therapeutics, University of Iowa, Iowa City, IA, United States; 4 Perspective Therapeutics, Seattle, WA, United States; 5 Department of Radiology, University of Iowa, Iowa City, IA, United States

**Keywords:** uterine cervical cancer, radiopharmaceutical, radiotherapy, fibroblast activation protein (FAP), Pb-212, Pb-203

## Abstract

Designing and interpreting early phase trials of theranostic radiopharmaceuticals remains challenging because it is difficult to isolate effective levels of activity in enriched patient populations that would be worth following up in later-phase development. This study explores the known challenges and emerging opportunities for clinical research on patients with uterine cervical cancer. We identified eight randomized combination trials for advanced-stage uterine cervix cancer that were activated between 2011 and 2022 and tabulated their results to determine whether combinations were better than individual constituents and which suitable study population is best situated for the study of a new theranostic agent. In this overview, we discuss exploitable vulnerabilities and radiobiology of cancer-associated fibroblasts, as these stromal cells are targets for nuclear and therapeutic radiation medicine. We also discuss investigational drugs that hold promise for the theranostic treatment of persistent, recurrent, or metastatic uterine cervical cancer, including inhibitors of fibroblast activation protein-alpha and ribonucleotide reductase. In our expert opinion, the development of a theranostic radiopharmaceutical should pursue the eventual goal of being tested in a randomized phase II monotherapy setting.

## Introduction

Phase II trials are conducted after phase I safety trials to provide preliminary evidence that an investigational therapy has adequate clinical activity for study in late phase III development (1). Single-arm phase II trials are generally avoided because these designs make it difficult to isolate the clinical activity of an investigational therapy from ancillary care. A randomized design is the preferred phase II option because of its unbiased comparison of treatments in well-characterized populations (1). Further refinement of the randomized design adds an enrichment strategy. Enriched phase II trials randomly allocate only biomarker-positive patients to an investigational therapy, thereby avoiding the treatment of biomarker-negative patients with therapies that are not expected to work for them (2). To avoid unnecessary participant exposure to radioactivity, the development of theranostic radiopharmaceuticals falls into an enrichment randomized phase II approach (3).

Metastatic uterine cervix cancer is rare among American women, with 2,004 [15%] of 13,360 women diagnosed with the disease in 2025 (4). One (19%) in five women survives five years (4), with some surviving only for six months (5). As there is more access to effective salvage regimens for later disease stages, randomized phase II trial designs with survival as the primary endpoint remain a challenge because the target population is large and trial completion time is long. This has led investigators to add prospective plans for clues to response and survival among expansion safety cohorts found in early phase trials, even though the data are regarded as too variable to draw reliable conclusions. One example where these notions were integrated is an early phase trial of [^203/212^Pb]Lead-PSV-359, a novel cyclic peptide targeting fibroblast activation protein-alpha (FAPα) (6), whose study population included women with previously treated genital neoplasms (NCT06710756, https://clinicaltrials.gov). Here, we use [^203/212^Pb]Pb-PSV-359 as an example of a trial design.

Herein, we discuss the challenges and opportunities of targeting FAPα in uterine cervical cancer imaging and treatment. To achieve this, we first discuss the impact of uterine cervix cancer disease presentation on the current levels of radiopharmaceutical clinical development. We then identified the essential properties of cancer-associated fibroblasts and their cell surface-expressed FAPα, focusing on immune modulation, treatment evasion, and radiobiology. Finally, we introduce concepts elemental to a theranostic radiopharmaceutical trial for second-line or more treatments of metastatic uterine cervix cancer, using the opportunities afforded by the United States National Cancer Institute (NCI) clinical trial enterprise as a means for possible development.

## Challenges and opportunities

### Impact of uterine cervix cancer disease presentation on radiopharmaceutical development

Abdominopelvic lymph node-positive uterine cervix cancer remains an aggressive initial disease stage that renders women at risk for persistent, recurrent, or metastatic disease. This disease often requires an initial radiation dose of 75+ Gy and radiosensitizing cisplatin chemotherapy (hereafter, radiochemotherapy) plus other chemoimmunotherapy to sterilize the disease (7–13). Immunotherapy or drug-conjugate therapy as single agents has been tested successfully in the later stages of the disease (14–17). Key elements from trials in these disease settings benchmark next-generation second- or third-line trials of theranostic radiopharmaceuticals and are briefly reviewed next for context.

Among the eight trials identified, two studies evaluated cytotoxic carboplatin-paclitaxel chemotherapy administered either before or after radiochemotherapy (**Table 1**). For example, the Australian-New Zealand Gynecologic Oncology Group (ANZGOG) OUTBACK phase III trial (2011–2017) randomly assigned 919 women with mostly stage ≤II (76%) disease to adjuvant or no further treatment (8). Five-year hazard for death was 0.90 (95% confidence interval [CI]: 0.70–1.17; 5-yr survival [OS]: 72 vs. 71%, *p* = 0.81), with more side effects noted after adjuvant chemotherapy. Four-month post-therapy metabolic complete response (mCR) on [^18^F]F-fluorodeoxyglucose (FDG) positron emission tomography (PET) was more frequent after adjuvant therapy (57%) than after standard therapy (50%, *p* = 0.14). A subsequent trial demonstrated the limited activity of adjuvant therapy in advanced-stage node-positive disease (18). Another example is the United Kingdom-led INTERLACE phase III trial (2012–2022), in which 500 women (stage ≤II 86%) were randomly allocated to induction chemotherapy for six weeks before radiochemotherapy (9). Three-year hazard for death was reduced by 40% (ratio: 0.60, 95% CI: 0.40–0.91; 3-yr OS: 85 vs. 80%, *p* = 0.02), despite the higher incidence of side effects observed in the induction chemotherapy arm. When considering both studies, the excess toxicity of chemotherapy around the radiochemotherapy treatment has lessened enthusiasm for additional combination studies, especially in stage II or earlier uterine cervical cancer, where radiochemotherapy alone is curative. Currently, these studies have prompted us to move away from the front-line setting for theranostic radiopharmaceutical development.

**Table 1:** Uterine cervical cancer disease stage and representation on select randomized phase III clinical trials.

Study (ref)	Disease setting	Treatment arm	DoT (weeks)	Any grade AEs of interest (%)	Distant relapse (%)	Efficacy	Reason for notability
GOG-0120 (7)	1st-line any node stage IIB (52%) or III-IVA (48%); node+ (14%) (n = 526)	RT-hydroxyurea (3 g m^−2^ MTh × 6) *v.* CRT-5FU-hydroxyurea (2 g m^−2^ MTh × 6) *v.* CRT	9 *v.* 9 *v.* 9	leukopenia (46 *v.* 53 *v.* 38)	10 *v.* 4 *v.* 3 *p* = NR	2-yr OS%: 60 *v.* 74 *v.* 74; HR 0.61 (0.44-0.81)	Randomized weekly cisplatin during radiotherapy
ANZGOG-0902 (8) [NCT01414608]	1st-line node+ stage 1B1 or any node IB2-II (76%) or IIIB-IVA (24%); node+ (48%) (n = 919)	CRT + carboplatin (AUC 5)-paclitaxel (155 mg m^−2^) q3wk × 4 *v.* CRT	24 *v.*8	anemia (66 *v.* 64), leukopenia (62 *v.* 71), neutropenia (41 *v.* 26)	13 *v.* 15 *p* = 0.12	2-yr OS%: 86 *v.* 85; HR 0.90 (0.70-0.1.17)	Randomized adjuvant chemotherapy after CRT
INTERLACE (9) [NCT01566240]	1st-line node+ stage 1B1 or any IB2-II (76%) or IIIB-IVA (24%); node+ (43%) (n = 500)	carboplatin (AUC 2)-paclitaxel (80 mg m^−2^) q1wk × 6, then CRT *v.* CRT	13 *v.* 6	anemia (59 *v.* 44), leukopenia (54 *v.* 28), neutropenia (58 *v.* 17)	7 *v.* 12 *p* = 0.02	2-yr OS%: 89 *v.* 86; HR 0.60 (0.40-0.91)	Randomized induction chemotherapy before CRT, allowed IMRT
NRG-GY006 (10) [NCT02466971]	1st-line any node stage IB2-IIB (72%) or IIIB-IVA (28%); node+ (55%) (n = 448)	triapine (25 mg m^−2^ MWF × 5) + CRT *v.* CRT	8 v. 8	anemia (54 *v.* 50), leukopenia (37 *v.* 42), conjunctivitis (5 *v.* 4)	NR	2-yr OS%: 88 *v.* 87; HR 1.0 (0.63-1.64)	Randomized RNR inhibitor during CRT, allowed IMRT
CALLA (11) [NCT03830866]	1st-line node+ stage IB2-IIB (34%) or III-IVA (66%); node+ (74%) (n = 770)	durvalumab (1,500 mg q4wks × 24) CRT *v.* placebo (q4wks × 24) CRT	67 *v.* 67	Thyroid disorder (6 *v.* 2), anemia (41 *v.* 40), leukopenia (14 *v.* 13), neutropenia (16 *v.* 13)	14 *v.* 18 *p* = NR	2-yr OS%: NR HR 0.78 (0.55-1.10)	Randomized PD-L1 blocking antibody in CRT, allowed IMRT
GOG-3047 (12, 13) [NCT04221945]	1st-line node+ stage IB2-IIB (44%) or III-IVA (56%); node+ (83%) (n = 1,060)	pembrolizumab (200 mg q3wks) CRT + pembrolizumab (400 mg q3wks × 15) *v.* placebo-CRT	51 *v.* 47	thyroid disorder (33 *v.* 7), anemia (59 *v.* 55), leukopenia (24 *v.* 17), neutropenia (21 *v.* 17)	NR	2-yr OS%: 87 *v.* 81; HR 0.67 (0.50-0.90)	Randomized PD-1 blocking antibody in CRT, allowed IMRT
GOG-3026 (14, 15) [NCT03635567]	1st-line stage IV; node+ (NR) (n = 617)	paclitaxel (175 mg m^−2^) + cisplatin (50 mg m^−2^) or carboplatin (AUC 5) × 6 + pembrolizumab (200 mg q3wks × 35) *v.* chemotherapy-placebo (± bevacizumab 15 mg kg^−1^)	56 *v.* 39	thyroid disorder (22 *v.* 11), anemia (41 *v.* 38), proteinuria (24 *v.* 14), neuropathy (27 *v.* 28)	ORR: 76 *v.* 62 *p* = NR	MedOS: 38 mo *v.* 23 mo HR 0.61 (0.47-0.80)	Randomized PD-1 blocking antibody during chemotherapy and antiangiogenic agent
GOG-3016 (16) [NCT03257267]	2nd or 3rd-line stage IV; node+ (NR) (n = 608)	cemiplimab (350 mg q3wks) *v.* choice chemotherapy	16 *v.* 12	anemia (25 *v.* 45), urine infection (12 *v.* 9), neutropenia (2 *v.* 15)	ORR: 16 *v.* 6 *p* < 0.001	MedOS: 12 mo *v.* 8.5 mo	Randomized PD-1 blocking antibody
GOG-3057 (17) [NCT04697628]	2nd or 3rd-line stage IV, node+ (NR) (n = 502)	tisotumab vedotin (2 mg kg^−1^ q3wks) *v.* choice chemotherapy	15 *v.* 11	anemia (23 *v.* 52), conjunctivitis (31 *v.* 0.4) neuropathy (28 *v.* 3)	ORR: 18 *v.* 5 *p* < 0.001	MedOS: 11.5 mo *v.* 9.5 mo	Randomized antibody-drug conjugate

AEs, adverse events; ANZGOG, Australia-New Zealand Gynecologic Oncology Group; CRT, weekly 40 mg m^−2^ × 6 cisplatin-based external beam radiotherapy followed by intracavitary brachytherapy; DoT, median duration of treatment; GOG, Gynecologic Oncology Group; HR, hazard ratio (95% confidence interval); IMRT, intensity-modulated radiation therapy; MedOS, median overall survival; n, number of participants; NR, not reported; ORR, objective response rate; PD-1, programmed cell death 1; ref, cited reference; RNR, ribonucleotide reductase.

One trial adopted a seamless phase II/III design to study an investigational triapine-cisplatin-radiation combination, whereby a randomized phase II efficacy signal was required to rationalize a larger phase III trial with a survival endpoint (10). In the 26-patient phase II part, the triapine-cisplatin-radiation arm had a higher three-month posttherapy PET mCR (92 vs. 82%, *p* = 0.58), which was sufficient for a ‘go’ decision for continued accrual to the phase III trial portion without a new master protocol document but a switch in the primary endpoint to overall survival (19). Typically, phase II patients contribute to phase III analysis, but not in this case. The open-label phase III trial (GY006, 2016–2022) ultimately studied 448 women (stage ≤II 72%) randomly assigned to the triapine-cisplatin-radiation combination or not, targeting a 10% gain in three-year survival in the investigational arm over the 72% control arm upper limit (**Table 1**) (10). At a pre-specified interim analysis, the three-year hazard for death was 1.02 (95% CI: 0.63–1.64; 3-yr OS: 80 vs. 78%, *p* = 0.78), determined not interesting enough to pursue in further development (despite many [216 [48%] of 448 participants] censored cases before the 3-year benchmark) (10). The disappointing rate of mCR (59 vs. 54%, *p* = 0.74) in this part of the study was also a surprise and remains difficult to interpret because of incomplete data (only 227 [51%] of 448 had results (10)). In our view, it would be a mistake not to notice that the control arm (78%) overperformed in regard to the null hypothesis target (72%) and historical data (68% (20),). We believe this is likely due to strict pre-therapy image-guided intensity-modulated radiotherapy (IG-IMRT) quality assurance (*i.e.*, 74% of patients had IG-IMRT expert review before actual treatment, likely improving radiation quality). Moreover, with another high (72%) stage ≤II disease proportion in a definitive phase III trial likely to be cured by radiochemotherapy alone, triapine-cisplatin-radiation yielded too few additional mCR responses to impact long-term overall survival. For these reasons, its oral formulation was deemed not worth pursuing for further development in a front-line uterine cervical cancer setting (21). All these observations left us unsatisfied with a front-line setting for an initial population to develop a theranostic agent for this disease.

Two trials investigated immunotherapy-radiochemotherapy combinations (**Table 1**). The CALLA double-blinded randomized phase III trial (2019–2020) accrued 770 women with either (a) staged IB2–IIB with regional node-positive disease (34%) or (b) staged III–IVA disease (66%) to receive either 24 cycles of durvalumab or placebo every four weeks (11). The hazard for death was 0.78 (95% CI: 0.55–1.10, *p* = not reported). Adverse events occurred in 62% of patients after durvalumab treatment, prompting one (13%) of eight patients to discontinue trial therapy. The KEYNOTE A18/GOG-3047 double-blind randomized phase III trial (2020–2022) studied 1,060 women with stage IB2–IIB with regional node-positive disease (44%) or stage III–IVA regardless of regional nodal status (56%), and administered five cycles of pembrolizumab or placebo every three weeks during radiochemotherapy followed, by 15 cycles of pembrolizumab or placebo every six weeks (12). Three-year hazard for death was reduced by 33% (0.67, 95% CI: 0.50–0.90; 3-yr OS: 83 vs. 75%, *p* = 0.004) (13). The hazard for disease progression or death was 0.72 (95% CI: 0.56–0.92) among women with programmed death ligand 1 (PD-L1)-positive (score ≥1) disease and 0.61 (95% CI: 0.18–2.1) among those with PD-L1-negative (score <1) disease (12). Attributable adverse events occurred in two-thirds of patients after pembrolizumab treatment, prompting 15% to stop treatment (12). Although improvement in survival was detected, faultfinders voice apprehension over this latter regimen’s one-year treatment duration that surpasses a willingness-to-pay threshold (22). Together, the irradiation-immunotherapy combination appears effective and suggests that the pairing of a theranostic therapeutic agent and immunotherapy may be intriguing in a node-positive advanced-stage uterine cervical cancer population.

Three trials offered retreatment designs, whereby entry criteria varied in the timing of progression of a participant’s disease after the prior line of treatment. One study involved a randomized phase III trial (2017–2020) of single-agent cemiplimab every three weeks for up to 96 weeks versus investigator-limited choice chemotherapy (16). The investigational arm was associated with a 31% reduction in the hazard of death (95% CI: 26%–44%, *p* < 0.001). The median progression-free survival was approximately 11 weeks after either treatment (hazard ratio: 0.75, 95% CI: 0.63–0.89, *p* < 0.001). The disease response to cemiplimab was 16% (vs. 6%, *p* < 0.01). The investigators recognized that the benefit with respect to the hazard for disease progression or death after cemiplimab treatment was made possible by the durable separation of the curves after the median intervals were reached (16). A second phase III trial (2018–2020) used paclitaxel-platinum chemotherapy for six cycles (with bevacizumab administered at the investigator’s discretion), plus allocation by random assignment, pembrolizumab, or placebo every three weeks for up to 35 cycles (14). There was a 39% (95% CI: 20%–53%) reduction in the hazard of death after the quadruplet (15). The disease response to the quadruplet therapy was 76% (vs. 62%, *p* = not reported) (15). As expected, the quadruplet was associated with a high (74%) clinically meaningful toxicity rate, with 41% of participants stopping treatment because of adverse events (15), precluding the wide adoption of the regimen. A third phase III trial (2021–2023) randomly allocated tisotumab vedotin every three weeks or limited-choice chemotherapy (17). There was a 30% reduction in the hazard of death (95% CI: 11%–46%, *p* = 0.004). The median progression-free survival was nearly 17 weeks after tisotumab vedotin and approximately 12 weeks after chemotherapy (0.67, 95% CI: 0.54–0.82, *p* < 0.001). The disease response to tisotumab vedotin was 18% (*vs.* 5%, *p* < 0.001). Criticisms of the tisotumab vedotin regimen focus on the agent’s high cost, potential for serious ocular toxicity and peripheral neuropathy, and the need for specialized ancillary care. To us, the persistent, recurrent, or metastatic disease setting is prime for theranostic radiopharmaceutical development, and the guiderails provided by these three trials should lead to an adequately powered randomized phase II trial for the initial evaluation of efficacy.

Therapeutic response depends on a combination of tumor molecular features, including somatic mutational and epigenetic landscapes, oncogene addiction, and tumor microenvironment. We recognize that metastatic uterine cervix cancer involves *de novo* (15% of all at initial diagnosis (4)) and metachronous metastases (up to 18% posttherapy (11)), which are genetically diverse with dissimilar prior radioimmunochemotherapy exposures. These factors lead to unpredictable responses and adverse events (14, 15), leaving ample room for investigating such phenomena using testable hypotheses in future radiopharmaceutical trials. Opportunities for this emerge in adequately powered single-arm phase II monotherapy trials (1).

### Essential radiobiological properties of cancer-associated fibroblasts

**Figure 1:**
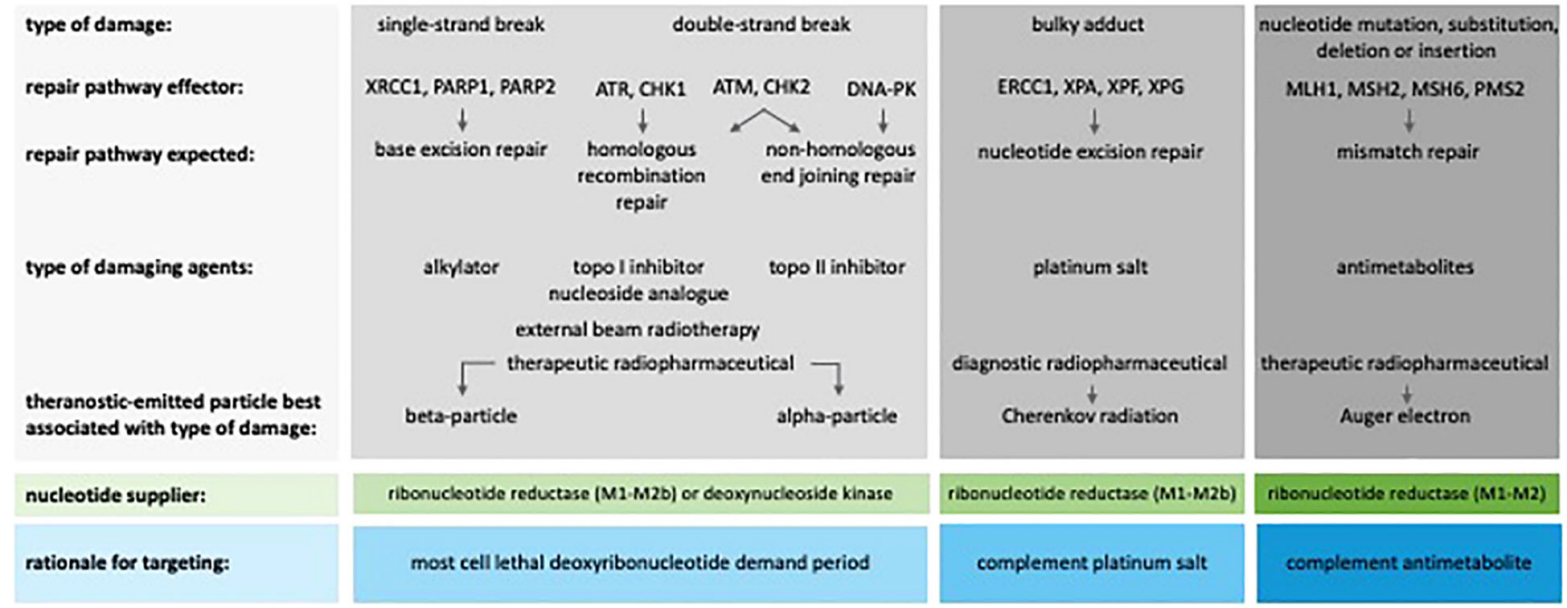
DNA damage response effectors and targets for theranostic radiopharmaceuticals, including a rationale for targeting these pathways. External beam radiotherapy may induce single- or double-strand breaks, thereby engaging multiple expected repair pathways. Due to the differing linear energy transfer of emitted particles from decaying therapeutic radionuclides, preferential recruitment of single-strand or double-strand repair is evident. ATM, ataxia-telangiectasia mutated; ATR, ataxia-telangiectasia and Rad3-related; CHK, checkpoint kinase; DNA-PK, DNA-dependent protein kinase; ERCC, excision repair cross-complementation group; MLH, mutL protein homolog; MSH, mutS homolog; PARP, poly(ADP-ribose) polymerase; PMS, postmeiotic segregation group; topo, topoisomerase; XP, xeroderma pigmentosum group; XRCC, X-ray repair cross-complementing protein.

Diverse forms of DNA damage evoke responses by a cell’s repair mechanisms, and while there is no absolute redundancy, backup repair pathways might compensate for abnormal or missing ones (23). In mammalian cells, there are five major repair pathways: (a) base excision repair for abasic sites, base-modified sites or single-strand breaks; (b) nucleotide excision repair for modified nucleotides; (c) mismatch repair for base-pairing replication errors; (d) homologous recombination repair (undamaged sister chromatid-requiring); and (e) nonhomologous end-joining repair (non-chromatid) for double-strand breaks. The response to radiation-induced nuclear DNA damage varies depending on the status of the molecular chokepoints (**Figure 1**). For example, G_1_/S-cell cycle phase checkpoint regulators, such as ATM, CHk2, and p53, stall signals for cell proliferation, activate ribonucleotide reductase (RNR, in its M1–M2b form) to increase deoxyribonucleotides (dNs), and subsequently permit repair of damaged DNA before DNA replication (24). At the intra-S cell cycle phase checkpoint, regulators such as ATR, CHK1, DNA-PK, and WEE1 delay the firing of replication origins, allowing already ramped-up RNR M1–M2 units to raise dN levels to facilitate DNA repair (25, 26). At entry into the G_2_/M cell cycle phase, effectors such as CHK1, WEE1, and MYT1 phosphorylate cyclin-dependent kinases, such as CDK1 (27), inactivating them to stall mitosis and promote RNR M2 proteolysis (28). Soluble factor crosstalk between cancer-associated fibroblasts and cancer cells represents a major opportunity to compensate for aberrant DNA damage responses.

One of the best-known disease-associated examples of cancer-associated fibroblast-cancer cell crosstalk occurs in uterine cervical cancer (29–35). Normal fibroblasts exhibit a spindle-shaped morphology, a mesenchymal lineage (expressing both α-smooth muscle actin [αSMA] for contractility and FAPα for sensing and responding to extracellular matrix [ECM] cues), and are responsible for ECM production, surveillance, and maintenance (36, 37). A cancer-associated fibroblast shares these essential properties but distinguishes itself from nearby cancer cells by *lacking* (a) epithelial, endothelial, and leukocyte markers and (b) abnormal DNA damage responses (37). Cancer-associated fibroblasts engage in a dynamic interface with nearby cancer cells to promote replicative immortality for tumor growth, refashion DNA damage responses through metabolic effects or soluble secreted factors, and evade antitumor immunity by disrupting macrophages and T-cell lymphocytes.

**Figure 2:**
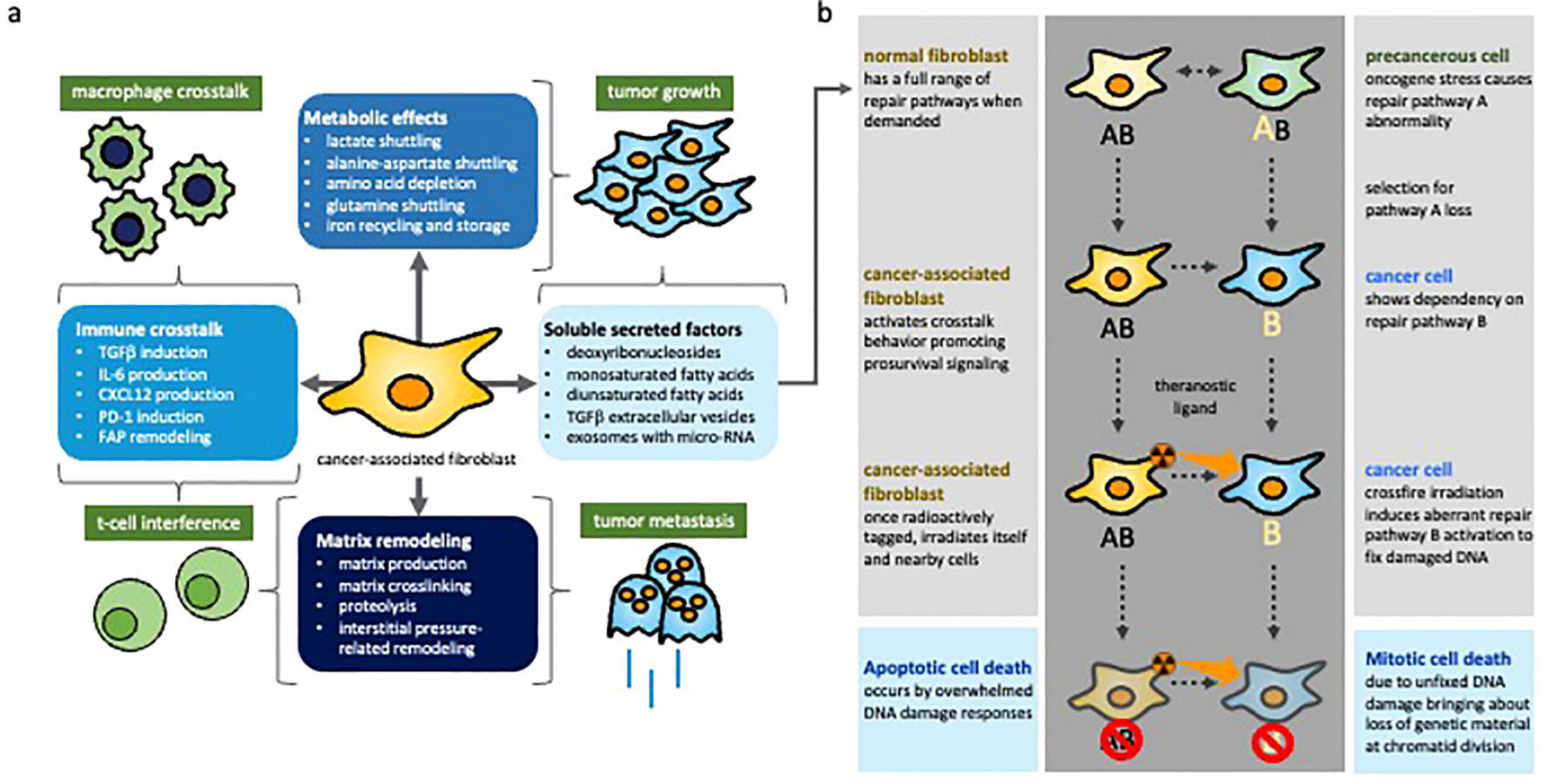
Essential properties of cancer-associated fibroblasts. **(A)** Dark green boxes indicate biological phenomena being governed, with light blue, gray, dark blue, and blue text boxes indicating the factors or mechanisms leading to process control. Brackets connect processes to the governed phenomena. Immune crosstalk manipulates macrophage and T cell activities. Matrix modeling and remodeling interfere with T-cell migration and metastasis potential. Both metabolic effects and soluble secreted factors result in tumor growth. **(B)** Despite a full complement of DNA repair pathways, theranostic ligand-tagged cancer-associated fibroblasts are sterilized due to high linear energy transfer, which disrupts nuclear DNA and causes cell death. Loss or aberration of DNA repair pathways leaves cancer cells vulnerable to crossfire irradiation and cell death due to failed processing of nuclear DNA damage.

**Figure 2** outlines the underlying concept and opportunities for theranostic radiopharmaceutical agents. The potential for human papillomavirus (HPV)-positive epithelial cells in intermediate and final degrees of neoplasia has been shown to promote a fibroblast-to-cancer-associated fibroblast transition, and these transitioned fibroblasts interact in a feedback loop to support replicative immortality in cancer cells (38). Deoxyribonucleosides leak into the interstitium of all mammalian cells (39) (including cancer-associated fibroblasts), allowing nearby proximate uterine cervical cancer cells to recover and use them to repair DNA (40). Cancer-associated fibroblasts also release free fatty acids (41), whose absorption alters cancer cell radiosensitivity through a p53 mechanism (42). Cancer-associated fibroblasts secrete transforming growth factor β (43), raising levels of epidermal growth factor receptor (EGFR (44),), which can confer radiation resistance (45). Efferent exosomes from cancer-associated fibroblasts transfer non-coding RNA such as miR-196a, to disrupt cyclin-dependent kinases (CDK), such as CDKN1B (or p27) (46), to alter master cell cycle controls, such as the CDK-phosphorylated retinoblastoma protein (pRb)-E2F transcription factor (E2F) axis. In this latter pathway, disrupted regulation of the CDK-pRb-E2F axis leads to unchecked E2F expression, resulting in downstream RNR M2 transcription origin firing (47). All these factors bestow pro-survival crosstalk signals. We hypothesize that a cancer-associated fibroblast, once tagged by a ligand or chelator carrying a therapeutic radionuclide, irradiates not only itself but also adjoining cancer cells by particle emission crossfire (**Figure 2**). We suggest that cancer cells harboring DNA damage response deficiencies have the potential for monotherapy lethality against tumor cells (or synthetic lethality, if paired with an inhibitor of a select compensatory repair pathway). If cancer-associated fibroblasts are sufficiently irradiated, they may die from overwhelming DNA damage responses.

The initial focus for DNA damage response inhibitors in uterine cervix cancer was in combination with antimetabolites or RNR-blocking chemotherapies (**Table 1**). The appreciation that immune modulators provide benefits has come to the forefront of such research, and a likely explanation can be found in a cell’s detection of free cytoplasmic genetic material (such as damaged fragments of nuclear DNA after irradiation or HPV viral DNA), which activates immunity against antigens. A link between cyclic GMP-AMP synthase (cGAS), an intrinsic DNA sensor, and the adaptor STING, an extrinsic immune response modifier, has been established (48). Moreover, it has been shown that HPV-E7 disrupts intrinsic antitumor immunity by inactivating STING via a Leu-X-Cys-X-Glu viral oncogene motif, like its inhibition of pRb (49). The regulation of cancer-associated fibroblasts by cGAS-STING in relation to afferent/efferent exosomes is an active area of research.

However, in the case of cancer-associated fibroblasts, FAPα expression plays a pivotal role in ECM modeling after irradiation (50). Mammalian FAPαs are composed of two identical subunits, housing an eight-bladed β-propeller domain and α/β-hydrolase domain (51) and localize in invadopodia (actin-rich cell protrusions) (52). FAPαs belong to the dipeptidyl peptidase family (similar to DPP IV). FAPαs digest denatured types I and III fibrillar collagen, reorienting the adhesion and migration of cancer cells and CAFs, leading to a denser and more tightly packed tumor microenvironment (TME) (53). This dense stroma can act as a physical barrier, increasing intratumoral pressure, collapsing blood vessels, raising hypoxia levels, and blocking access to anticancer drugs (54). While undetectable in quiescent fibroblasts and epithelial cells, FAPα is typically expressed in embryonic stromal tissues, healing wounds, and uterine scars (51, 55, 56). Up to 67% of women show menstrual fibroblast activation, such that FAP-directed agents show uptake in the endometrium, a phenomenon that decreases with age (57). However, FAPα is overexpressed in the tumor stroma of breast, cervical, colorectal, gastric, lung, pancreatic, prostate, thyroid, and urothelial cancers (29, 58). FAPα expression is restricted to the cancer cell-cancer-associated fibroblast interface, and FAPα inhibitor-linked nuclear medicine agents can distinguish uterine cervix cancer cells metastasizing to lymph nodes from metabolically immune-reactive lymph nodes, where [^18^F]F-FDG positron emission tomography is ineffective (30–35). Further data on FAPα are emerging from active preclinical and clinical research, and good summaries of this biological entity can be found elsewhere (36, 59, 60). Therefore, we next turn to the opportunity of targeted FAPα inhibitors (FAPI) as next-generation theranostic radiopharmaceuticals.

## Perspective on theranostic radiopharmaceuticals for uterine cervical cancer

### Targeted theranostic radiopharmaceuticals for uterine cervical cancer

Positron emission tomography using the molecular radioactive tracer 2-[^18^F]-fluoro-2-deoxy-D-glucose ([^18^F]F-FDG), an analog of glucose trapped by hexokinase, has emerged as a sufficiently discriminating noninvasive imaging method for trial use to detect initial regional or metastatic uterine cervical cancer (10). A published long-term series of 402 women showed that [^18^F]F-FDG PET remains useful for monitoring the treatment effect and detecting persistent, recurrent, or metastatic disease (61). However, the accuracy of [^18^F]F-FDG to accurately diagnose and monitor therapeutic response in nodal tissues has come into question due to the failure of [^18^F]F-FDG to discriminate between inflammatory reactions and hypermetabolic cancer (61). This gap led to the study of noninvasive radiolabeled FAPI agents as opportune highly discerning agents capable of improving the accuracy of positron emission tomography in cancers of the uterine cervix, due to the substantial cancer-associated fibroblasts exclusively expressing the FAPα marker in its tumors, regardless of its primary, nodal, or metastatic site. FAPI agents can be loaded with both diagnostic and therapeutic element-identical radionuclides, widening their therapeutic index. The agent FAPI-46 stimulated early studies of nuclear medicine in uterine cervical cancer patients, as it carries [^68^Ga]Gallium as a beta-positive emitting radiotracer linked to an FAPα-targeting quinoline structure, or [^90^Y]Yttrium or [^177^Lu]Lutetium as a therapeutic radionuclide (30–36).

Superior diagnostic performance of [^68^Ga]Ga-FAPI-46 positron emission tomography-computed tomography (PETCT) compared with [^18^F]F-FDG PETCT has been attributed mainly due to higher tumor-to-background ratios. High uptake has been detected in 28 malignant tumors of epithelial and sarcomatous types, regardless of metabolic glucose activity (often influential in FDG PET) (31). A prospective study involving 35 patients previously diagnosed with breast (n = 9, 26%), ovarian (n = 10, 29%), or uterine cervical (n = 16, 45%) cancers explored the potential benefits of [^68^Ga]Ga-FAPI PETCT imaging compared with [^18^F]F-FDG PETCT for detecting lesions (35). Detection of untreated primary tumors was accurate in 85% (23 of 27) of cases for [^18^F]F-FDG PETCT and 100% (27 of 27) of cases for [^68^Ga]Ga-FAPI PETCT. In a disease spread to lymph node analysis, sensitivity (86% [31 of 36] vs. 97% [35 of 36]), specificity (66% [23 of 35] vs. 100% [35 of 35]), and accuracy (80% [54 of 71] vs. 94% [67 of 71]) were all improved after [^68^Ga]Ga-FAPI PETCT. However, additional research on FAPI theranostic radiopharmaceuticals is needed.

**Figure 3:**
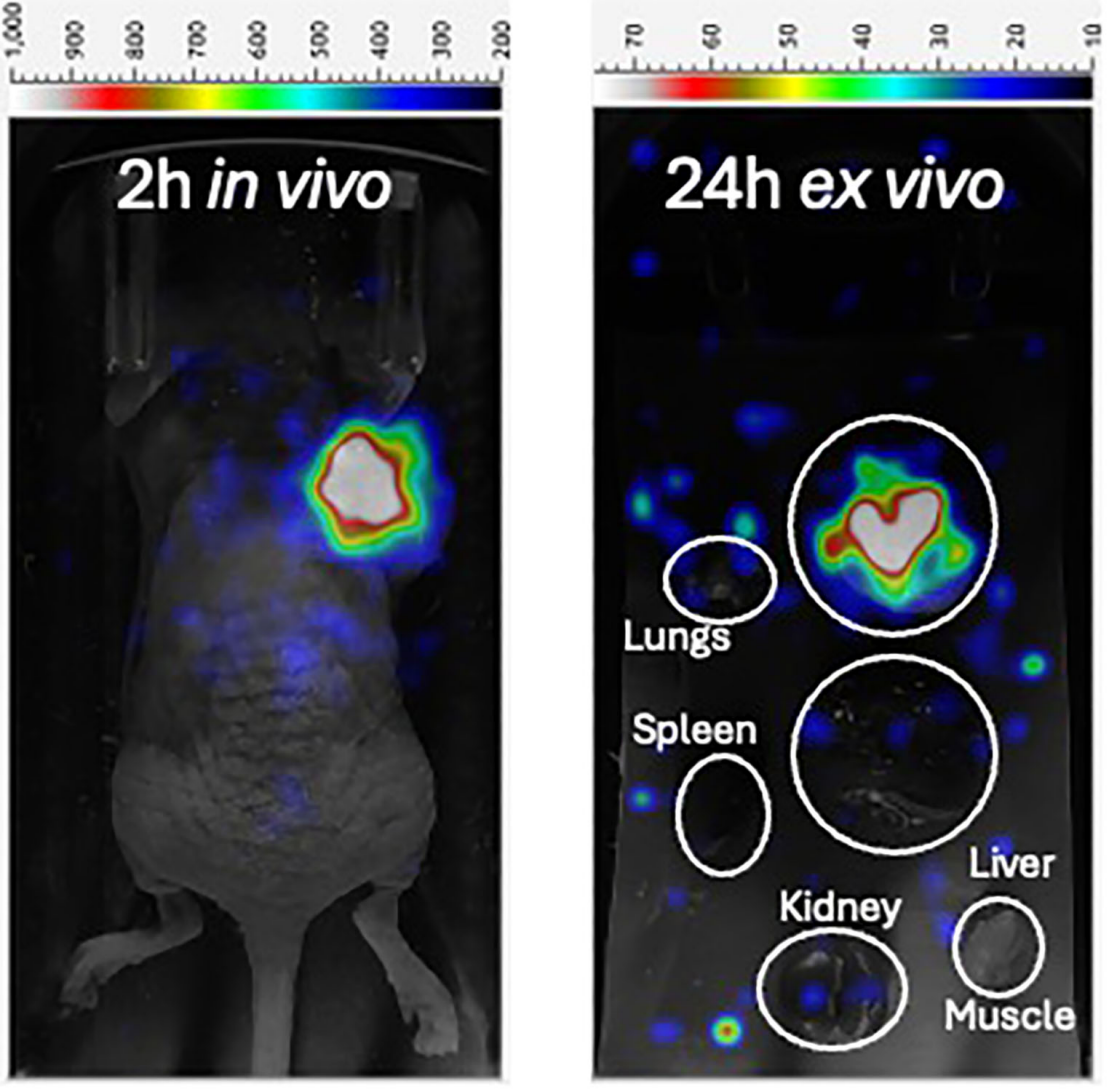
Planar imaging of [^212^Pb]Pb-PSV-359 (1.2 MBq) in a female athymic nude mouse bearing HT1080-hFAP xenograft. Single-photon emission computed tomography (SPECT) images acquired at 2 h *in vivo* and 24 h *ex vivo* revealed strong [^212^Pb]Pb-PSV-359 tumor uptake and retention. There is little to no vital organ uptake or retention. This research was originally published in the *Journal of Nuclear Medicine* (6). © SNMMI.

Currently, the use of element-identical theranostic pairs (same radioactive-decaying element, different isotopes such as [^203/212^Pb]Lead) for nuclear imaging and radiation medicine offers several advantages, including minimizing alternatives in chemical behavior and simplifying synthesis, which ultimately allows for a more accurate evaluation of therapeutic efficacy using *in vivo* body imaging. ^203^Pb (SPECT imaging; T½ = 52 h) and ^212^Pb (alpha-particle therapy; T½ = 11 h) are gradually being used in element-identical theranostic pair drug discovery. Phage display with site-specific cyclization led to the discovery of an optimal candidate molecule, PSV-359 (6). To date, preclinical studies have shown a superior binding affinity (K_d_ = 1.8 nM) and specificity (K = 0.4 nM) of PSV-359 against human FAPα, with no unnecessary binding to prolyl endopeptidase or DPP IV, which is usual of the drug class. The serum half-life stability of [^203^Pb]Pb-PSV-359 was 96 h. In a fibrosarcoma xenograft model (HT1080 expressing human FAPα), high tumor uptake (20% ID/g at 1.5 h, 14% ID/g at 24 h) and fast blood clearance (0.2%ID/g at 3 h) by the kidneys (4.7%ID/g at 24 h) were observed (6). The in-residence time for tumors was high in [^203/212^Pb]Pb-PSV-359 studies of athymic nude mice bearing HT1080-h FAPα tumors, with 80% complete responses noted at the 90-day survival endpoint. No hematologic adverse events were recorded. **Figure 3** shows proof-of-concept images of an animal model (6).

**Figure 4:**
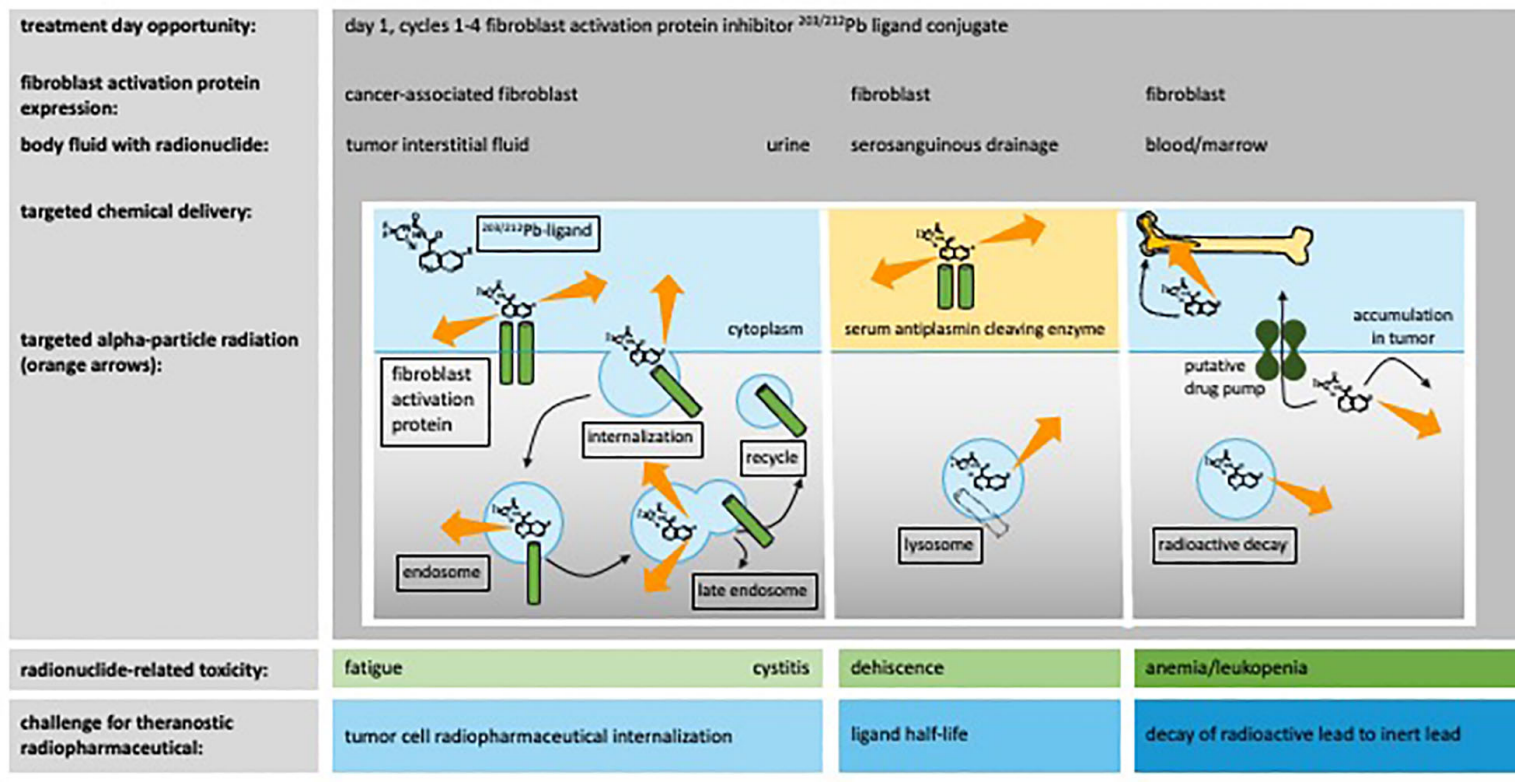
Fibroblast activation protein-alpha inhibitor theranostic radiopharmaceuticals. A fibroblast activation protein-alpha-targeted theranostic quinoline agent and its off-target normal organ toxicities were charted in relation to ^203/212^Pb radionuclide delivery and adverse events (toxicities) of special interest. The tumor interstitial fluid, urine, wound serosanguinous drainage, and blood/marrow show molecular expression of fibroblast activation protein-alpha (or its serum equivalent antiplasmin cleaving enzyme [APCE]) and are listed together that might have demonstrable levels of off-target radiopharmaceutical localization. The mechanistic steps of receptor processing likely involved in the intended irradiation of tumor cells or in unintended toxicity of normal cells are marked in boxes. The challenges for radiopharmaceuticals are highlighted in blue boxes.

Theranostic chemistry requires high affinity target binding in cancer-associated fibroblasts and low affinity for normal body cells to achieve a theoretically high therapeutic window. We believe that it is desirable for radiolabeled ligands to seek surface antigens before caveolin-dependent internalization and endosomal degradation. This permits the crossfire of decaying particles to transfer their linear energy to resident and nearby cells, ablating both cancer-associated fibroblasts and cancer cells. **Figure 4** illustrates our thoughts on this issue. Elimination of cancer associated fibroblasts might have the unintended benefit of improving anticancer drug delivery by eradicating blocking cells, reducing interstitial pressure, improving lymphocyte infiltration, and lessening the pro-survival TME (**Table 2**). We believe that the advantage of FAPI-based theranostics lies in the observation that normal cells rarely express the target antigen, whereas cancer-associated fibroblasts do. However, since FAPα is expressed in healing wounds and uterine scars, this would likely exclude women with fresh postsurgical incisions or active liver fibrosis ineligible for therapy (**Figure 4** summarizes some of these risks). Indeed, “hot” pharmaceutical ligands might have unwanted off-target biological effects that reflect nonspecific or inappropriate target antigen recognition in normal cells and might manifest as toxicity of special interest. In the example of the [^203/212^Pb]Pb anti-FAPα theranostic pair, off-target pharmaceutical toxicity remains to be characterized; a phase I trial of [^203/212^Pb]Pb-PSV-359 should provide this clinical context (NCT06710756).

**Table 2:** Putative uterine cervical cancer tumor microenvironment effectors and radiosensitivity.

Cell type	Essential properties	RNR status (ref)	D0* (ref)
Tumor-associated macrophage	Modulates T-cell lymphocytes Provides anticancer drug resistance	Low, Inducible (65)	10 Gy (66)
Tumor-associated dendritic cell	Presents antigens to lymphocytes	Low, Inducible (65)	10 Gy (67)
Myeloid-derived suppressor cell	Suppresses antitumor immune response Upholds immune evasion	Low, Inducible (65)	10 Gy (67)
B-cell lymphocyte	Aids in cytotoxic immune responses	Low, Inducible (68)	1.5 Gy (67)
T-cell lymphocyte	CD8+ cytotoxic and CD4+ helper immunity Restricts tumorigenesis	Low, Inducible (69)	0.5 Gy (67)
Neutrophil	Stimulates inflammation Induces immunosuppression	Low, Inducible (69)	0.8 Gy (70)
Adipocyte	Regulation of tumoral energy metabolism Promotion of tumor cell progression	Very Low, Dormant (71)	7 Gy (72)
Resting G_0_/G_1_ fibroblast	Produces extracellular matrix Activate upon tissue injury	Low, Inducible (24)	4 Gy (73)
Cancer-associated fibroblast	Remodels extracellular matrix Upholds immune evasion Promotes replicative immortality Refashions DNA damage responses	20-fold High, Active (24)	5 Gy (74)
Uterine cervix cancer cell	Sustained proliferative signaling Evasion from tumor immunosuppression Resistance to cell death Replicative immortality Reprogrammed energy metabolism Induction of angiogenesis Activation of invasion and metastasis	Up to 100-fold High, Overactive (75)	6 Gy (76)

Gy, gray; ref, cited reference.

*When cells are exposed to radiation, some survive and some die. A cell survival curve plots the fraction of cells that survive (surviving fraction) against the radiation dose exposure. The D0 value is a characteristic parameter of the cell survival curve, specifically the dose required to reduce the surviving fraction to 37% (approximately 1/e). A lower D0 value indicates that a cell population is more sensitive to radiation, meaning it takes a smaller dose to reduce the surviving fraction to 37%.

Up to 34% of women with uterine cervical cancer report urinary incontinence (62), raising the risk of cutaneous radiation injury, infection, pain, or unintentional radioactivity exposure to others, which is a concern when undergoing therapy with kidney-filtered radiopharmaceuticals excreted in the urine, such as [^212^Pb]Pb. Graded urinary incontinence (grade 1: occasional; grade 2: spontaneous; grade 3: intervention indicated [e.g., clamp or catheter]) might guide satisfactory urine collection strategies on the day of agent administration (grade 1: pads or briefs; grade 2: external catheter; grade 3: indwelling catheter) (63).

Cellular expansion, necessary for the daily maintenance of more than 400 billion blood cells, renders the hematopoietic system particularly sensitive to radiation injury, even after low-dose irradiation exposure (64). Total marrow irradiation manifests as profound acute peripheral cytopenias, subacute reduction in hematopoietic progenitor cells, and late marrow adiposity, which affect hematopoietic recovery (64). For these reasons, under conditions of [^212^Pb]Pb exposure, four weeks of anemia/leukopenia are expected. Currently, effective interventions are limited to pharmaceuticals and transfusions.

**Figure 5:**
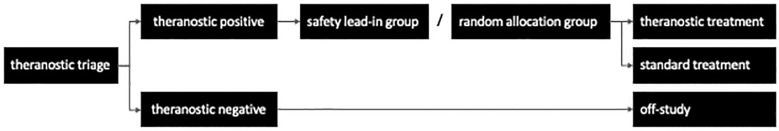
Theranostic radiopharmaceutical target-driven randomized phase II trial design isolating treatment effect. This enrichment approach evaluates a diagnostic radionuclide in an element-identical theranostic pair as a triage step for all the trial participants. Only theranostic-positive patients in whom the target has been verified proceed to therapeutic radionuclide treatment. An optional safety lead-in group of disease-specific participants ensures the tolerance of therapeutic radionuclide treatment if early phase trials are tumor-agnostic studies. Otherwise, random allocation of therapeutic radionuclide treatment was applied only to theranostic-positive participants.

**Figure 5** shows our suggested randomized phase II trial approach for a theranostic radiopharmaceutical agent. In our phase II study, a theranostic imaging triage step (using a diagnostic radiotracer to enrich participants expressing the target) segregated participants into theranostic-negative and theranostic-positive groups. Theranostic-negative participants contribute data on the performance of the diagnostic radiotracer but are regarded as off-study for protocol-specified treatments. Theranostic-positive participants provided data on the performance of the diagnostic radiotracer and proceeded to all therapeutic parts of the trial. As an optional step, a safety lead-in part involves a small number of participants receiving treatment first to identify and address potential safety issues or adverse reactions, ensuring that the trial can proceed safely within the main population. The theranostic randomized step allocates a therapeutic radionuclide (such as [^212^Pb]Pb-PSV-359 against cancer-associated fibroblast FAPα) for protocol-specified experimental treatment or standard treatment. Such a design isolates the clinical activity of investigational therapies. If a second- or third-line persistent, recurrent, or metastatic uterine cervical cancer population is selected for the study, then a survival primary endpoint would be appropriate.

## Conclusion

In summary, this study discusses the overall vision for FAPI radiopharmaceutical clinical development in relation to its application in uterine cervical cancer patients. It reviews the available evidence for cancer-associated fibroblast evasion of anticancer immune effectors, promotion of cancer cell replicative immortality, and reshaping of relevant cancer cell DNA damage responses. It also offers a perspective on an early phase radiopharmaceutical clinical trial demonstration project for women with persistent, recurrent, or metastatic uterine cervical cancers requiring second-line or higher treatment. We predict that FAPI-based theranostic agents may be a preferred treatment approach over immunochemotherapy for women with such diseases.
